# Comprehensive Analysis of Strong Opioid Side Effects in Palliative Care Using the SIDER Database

**DOI:** 10.3390/jcm14051410

**Published:** 2025-02-20

**Authors:** Risako Hirai, Motoki Kei, Yoshihiro Uesawa

**Affiliations:** 1 Department of Medical Molecular Informatics, Meiji Pharmaceutical University, 2-522-1 Noshio, Kiyose, Tokyo 204-8588, Japan; 2 Department of Pharmacy, Tokyo Women’s Medical University Adachi Medical Center, 4-33-1 Kohoku, Adachi-ku, Tokyo 123-8558, Japan

**Keywords:** strong opioids, opioid switching, side effect profile, SIDER database, palliative care, principal component analysis, cluster analysis

## Abstract

**Background/Objectives**: There exist multiple opioid-based treatments in palliative care, each with distinct side effect profiles. When adverse events occur, switching opioids can help maintain effective pain management. However, owing to limited clinical evidence, no comprehensive guidelines exist for opioid switching. This study employed the Side Effect Resource (SIDER) database, which aggregates adverse event data from clinical trials and package inserts, to analyze the side effects of five commonly used “strong opioids” in palliative care in Japan, namely morphine, fentanyl, oxycodone, hydromorphone, and tapentadol. **Methods**: Data on the names and incidence of adverse events for each opioid were extracted from SIDER 4.1, developed by the Max Delbrück Center for Molecular Medicine. Cluster analysis and principal component analysis were performed to interpret the data. **Results**: The key side effects of opioids were nausea, vomiting, constipation, and drowsiness. Fentanyl was more frequently associated with nausea and vomiting but less frequently with constipation and drowsiness. Tapentadol caused nausea relatively more frequently and constipation less frequently. Oxycodone was prominently linked to drowsiness, whereas morphine was frequently associated with constipation and drowsiness. Hydromorphone was associated with higher rates of constipation and vomiting but lower incidences of nausea and drowsiness. **Conclusions**: All side effects characterizing the opioids were related to μ-opioid receptor stimulation, although the present study findings highlight differences in the frequency of specific side effects among the opioids. These results provide objective insights that can guide opioid switching in response to adverse effects.

## 1. Introduction

In palliative care, opioids are essential for improving patients’ quality of life by managing pain, particularly cancer-related pain. Their analgesic effects stem from agonist activity at the μ-opioid receptor (MOR) in the central nervous system [1]. However, MORs are also expressed in peripheral organs, wherein their activation can lead to diverse side effects. For example, stimulation of MORs in the respiratory center can cause respiratory depression, whereas activation in the chemoreceptor trigger zone induces nausea and vomiting [2,3]. Additionally, opioids’ central effects can result in drowsiness, delirium, and somnolence [4,5]. Although these side effects are consistently associated with MOR agonist activity, their frequency varies markedly among different opioids [6]. For instance, morphine frequently causes constipation and drowsiness, fentanyl has a higher tendency to induce nausea or vomiting, and oxycodone can lead to pronounced sedation. Hydromorphone is often associated with gastrointestinal side effects such as vomiting and constipation, whereas tapentadol is associated with a generally lower incidence of constipation. Therefore, when patients experience adverse events during opioid therapy, switching to another opioid is often necessary to maintain their quality of life. Monitoring and managing these symptoms [7,8,9,10,11,12,13,14] are critical aspects of clinical practice. However, owing to the limited comprehensive research on opioid-induced side effects, opioid switching is often guided by insufficient evidence.

In this study, we analyzed the side effects of “strong opioids” commonly used in palliative care in Japan using data from the Side Effect Resource (SIDER) database [15]. SIDER, which has been developed by the European Molecular Biology Laboratory in Germany and is accessible online [16,17], compiles detailed data on drug side effects and is widely used in pharmaceutical research and drug safety evaluation. The latest version, SIDER 4.1, released on 21 October 2015, includes data on 1430 drugs. SIDER is a valuable resource for researchers and healthcare professionals, offering critical insights into the risks of side effects and facilitating the prediction of drug-drug interactions. It integrates adverse event data from clinical trials, package inserts, and the U.S. Food and Drug Administration. Notably, SIDER includes adverse event incidence data derived from clinical trials and package inserts, providing an analytical advantage over spontaneous reporting databases, such as VisiBase, FAERS, and JADER, which lack data on routine drug use cases, hindering the calculation of incidence rates.

A prior study based on SIDER analyzed clinical events related to clozapine treatment and compared side effect data from SIDER with electronic health records [18]. This study assessed the consistency among different text-mining methods based on electronic health records and validated SIDER’s utility. It highlighted SIDER’s role in assessing real-world data that corroborate previous study findings.

In summary, SIDER offers major advantages for understanding drug side effects, including their frequency and severity. Using SIDER to compare the safety profiles of different opioids, the present study aimed to provide valuable insights for guiding opioid selection during switching and related treatments. Specifically, this study highlights the distinct side effect profiles of five strong opioids, namely morphine, fentanyl, oxycodone, hydromorphone, and tapentadol, providing objective insights to guide opioid switching and optimize pain management in clinical settings, ultimately improving therapeutic options.

## 2. Materials and Methods

### 2.1. Selection of Target Opioids

Using SIDER 4.1, we extracted data on strong opioids used in palliative care in Japan [15]. Based on the Guidelines for the Management of Cancer Pain [19] by the Japanese Society for Palliative Medicine, we identified morphine, fentanyl, oxycodone, hydromorphone, tapentadol, and methadone as strong opioids for this analysis.

### 2.2. Incidence Rates of Side Effects Associated with the Target Opioids

SIDER contains data on 1430 drugs and 5880 side effect names, yielding 140,064 drug and side effect pairs [15]. Incidence data for each drug-side effect combination are derived from package inserts and the literature. Drugs listed in SIDER are uniquely identified by a compound identifier (CID) assigned by the PubChem database of the U.S. National Institutes of Health. Linking the drug name table to the side effect frequency table via the CID enables evaluation of the incidence of side effects for each drug.

SIDER’s side effect frequency table categorizes incidence rates as *very common*, *very frequent*, *common*, *frequent*, *uncommon*, *infrequent*, *rare*, and *very rare*, corresponding to a 0–1 range. For instance, *very common* and *very frequent* indicate an incidence range of 0.1–1, *common* and *frequent* indicate 0.01–1, *uncommon* and *infrequent* indicate 0.001–0.01, *rare* indicates 0–0.001, and *very rare* indicates 0–0.0001. Some incidence rates in the 0–1 range are provided as numeric values. Approximately 52% of the drugs listed in SIDER include incidence data [15]. However, the diverse sources of these incidence data, encompassing different clinical trials and severity levels, introduce variability that must be accounted for. To enable comparisons across opioids, we standardized the incidences of side effects for cluster analysis and principal component analysis (PCA), which are described in Section 2.4 and Section 2.5, respectively.

### 2.3. Calculation of Side Effect Incidence Rates for Target Opioids

The side effect names in SIDER 4.1 are mapped to the Medical Dictionary for Regulatory Activities (MedDRA). In this study, we used the lowest level term (LLT) in MedDRA version 16.1 to define side effect names.

We extracted the target opioids from SIDER and obtained the lower and upper bounds of the incidence rate for each LLT linked to these opioids, calculating the mean value of these bounds. For example, for the *rare* category, the lower bound, upper bound, and mean were 0, 0.001, and 0.0005, respectively. Specifically, for each frequency category, we used the following formula to calculate the mean incidence rate:

Mean incidence rate (%) = {(LowerBound + UpperBound)/2} × 100

If multiple sources reported the same side effect for a given opioid, we considered the median of the mean values. Finally, we applied a base-10 logarithmic transformation to the resulting percentages:

log_10_(Mean incidence rate (%))

This transformation helped normalize the data for subsequent clustering and PCA.

### 2.4. Cluster Analysis

Based on the incidence of the target opioids’ side effects, we performed hierarchical cluster analysis using Ward’s method [20]. Initially, we extracted all side effect names with reported incidence for each opioid studied. Cluster analysis was then conducted to compare side effect incidence rates across opioids. Before clustering, the incidence rates were standardized, setting the mean and variance to 0 and 1, respectively.

### 2.5. PCA

Based on the results of cluster analysis, we performed PCA [21] of the side effects identified as having higher risks among the target opioids. PCA reduces dataset dimensionality to identify principal component axes that best explain variance, enabling efficient data visualization and interpretation. By plotting the score plot and loading vectors for each opioid and side effect, we interpreted the principal components and clarified relationships between drugs and side effects. This analysis was conducted using the correlation matrix.

### 2.6. Statistical Analysis

Data handling in the SIDER database, cluster analysis, and PCA were conducted using JMP Pro 18.0 (SAS Institute Inc., Cary, NC, USA).

## 3. Results

### 3.1. Target Opioids and Side Effects

The strong opioids approved for use in Japan are morphine, fentanyl, oxycodone, hydromorphone, tapentadol, and methadone. However, owing to the lack of side effect incidence data for methadone in SIDER, we focused only on morphine, fentanyl, oxycodone, hydromorphone, and tapentadol. For these five opioids, we extracted all side effect names and their corresponding incidence data from SIDER, yielding 191 side effect names (Table S1). Among these, only 10 side effects were commonly listed across all 5 target opioids: nausea, dizziness, headache, somnolence, vomiting, constipation, dry mouth, hyperhidrosis, pruritus, and asthenia. The remaining 181 side effects lacked incidence data for at least one opioid. Thus, the 10 side effects, for which data were available across all studied opioids, were selected for further analysis, as they were the most useful for profiling differences in side effects. Accordingly, cluster analysis and PCA were conducted based on the incidence of these 10 side effects.

### 3.2. Cluster Analysis

**Figure 1:**
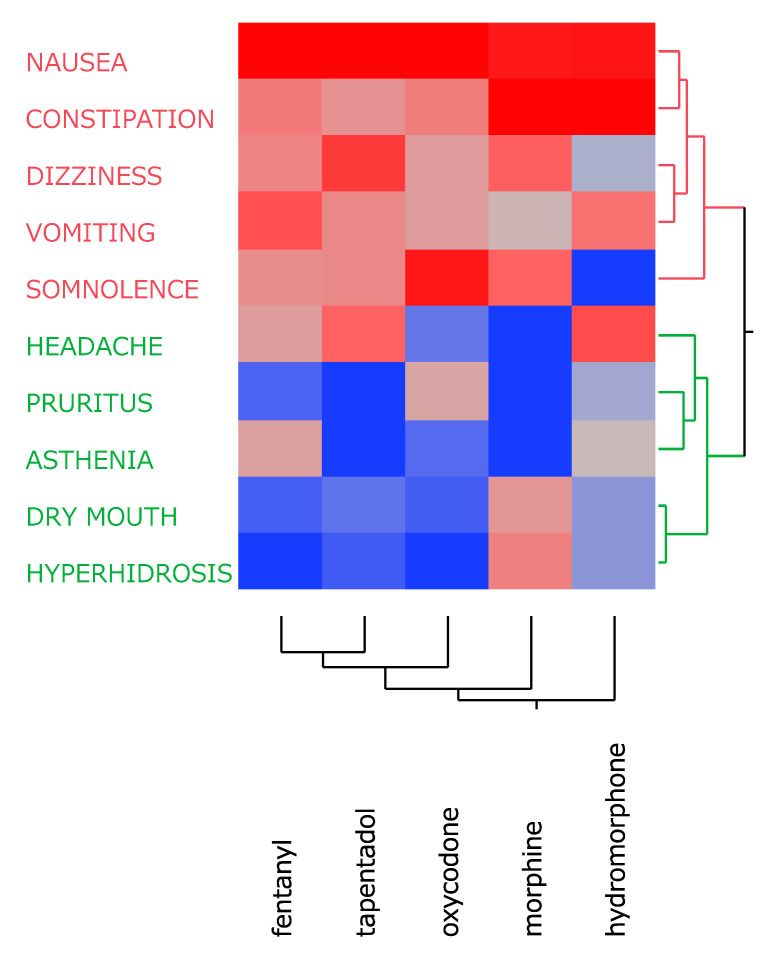
Cluster analysis based on side effect incidence rates among the target opioids. Side effects were grouped into two clusters (red and green) based on their incidence rates across opioids. Vertical and horizontal axes represent side effect names and opioid names, respectively. Deeper red indicates higher incidence rates, whereas deeper blue corresponds to lower incidence rates.

Using a cluster analysis based on the incidence rates of side effects, the abovementioned 10 side effects were categorized into two distinct clusters (Figure 1). Cluster 1 (red) included side effects with relatively high incidence rates across all opioids (nausea, constipation, dizziness, vomiting, and somnolence). Cluster 2 (green) comprised side effects with relatively low incidence rates (headache, pruritus, asthenia, dry mouth, and hyperhidrosis). These classification results were further explored using PCA to better understand the relationships among these side effects and highlight the distinguishing characteristics of each opioid.

### 3.3. PCA

**Figure 2:**
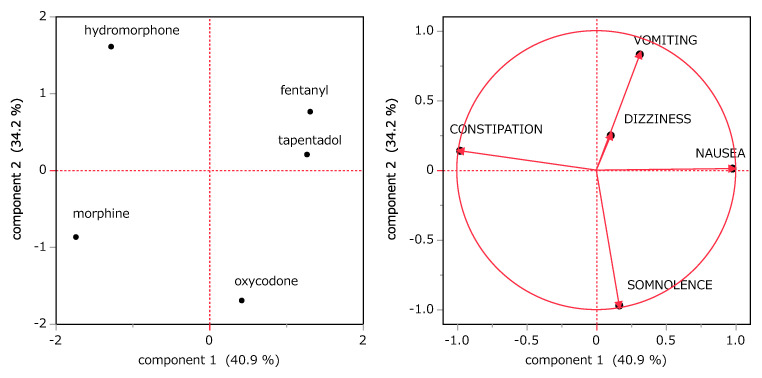
Principal component analysis of the target opioids and their major side effects. Left panel: score plot; right panel: loading vectors. Horizontal and vertical axes represent the first and second principal components, respectively.

Based on cluster analysis, five side effects (nausea, constipation, dizziness, vomiting, and somnolence) were identified as having relatively high incidence across all opioids. These side effects were further examined using PCA (Figure 2). The first and second principal components explained 40.9% and 34.2% of the total variance, respectively, for a cumulative total of 75.1%. The loading vectors revealed that nausea, vomiting, constipation, and somnolence were strongly associated with these principal components. Based on the score plot and loading vectors, the analysis highlighted distinct opioid-side effect relationships. Fentanyl was associated with a higher incidence of nausea and vomiting compared with other opioids, whereas tapentadol showed a relatively strong tendency for nausea. Oxycodone was prominently associated with somnolence, whereas morphine was more likely to induce both constipation and somnolence. Hydromorphone caused constipation and vomiting more frequently than other opioids.

## 4. Discussion

The five strong opioids investigated in this study, namely morphine, fentanyl, oxycodone, hydromorphone, and tapentadol, constitute the drugs of choice for palliative care in Japan, particularly for managing severe cancer-related pain. In clinical practice, opioid switching, i.e., adjusting the opioid based on the patient’s clinical status, is an important strategy for optimizing analgesia and side effect management [22]. For example, if an opioid causes severe constipation, switching to an alternative with a lower incidence of this side effect may improve the patient’s condition. This approach can be similarly applied to other side effects. Despite the wide variety of side effects reported for different opioids, only a few studies have comprehensively compared them. For instance, the “Guidelines for the Pharmacological Treatment of Cancer Pain (2020 Edition)” [19] from the Japanese Society for Palliative Medicine has stated that tapentadol is associated with lower incidences of constipation, vomiting, and neurological symptoms than oxycodone or morphine [23,24,25]. However, this finding provides only a limited perspective on the broader side effect profiles of strong opioids. In contrast, the present study used the SIDER database to comprehensively analyze the incidence rates of major side effects for five strong opioids, providing objective data to support opioid switching decisions.

Because the incidence data for opioids in SIDER are compiled from various clinical trials and literature sources, they may vary in dosage and severity levels across study populations [15,16]. For example, morphine does not exert a clear ceiling effect for its analgesic action, and higher doses may enhance pain relief [26]. As such, datasets containing high-dose cases may exhibit higher incidences of side effects. To address this, we standardized the incidence rates for each opioid during cluster analysis and PCA, allowing for more meaningful cross-opioid comparisons.

### 4.1. Comparison with Previous Studies by Side Effect Category

#### 4.1.1. Nausea/Vomiting

Cluster analysis categorized the 10 side effects into high- and low-incidence groups. In the high-incidence group, nausea and vomiting were common across all five opioids [6,27,28], with these side effects being especially problematic in chronic pain management, as they can reduce therapeutic effectiveness [27]. Nausea and vomiting triggered by central and peripheral MOR stimulation can result in major discomfort for patients [6,28]. In the current study, PCA showed that fentanyl and tapentadol were more likely to induce nausea and vomiting. Previous studies have reported an increased risk of vomiting within 24 h of fentanyl administration [29,30] and a higher incidence of nausea and vomiting during double-blind maintenance periods with tapentadol [31], aligning with our findings.

#### 4.1.2. Constipation

Constipation is primarily caused by the peripheral effects of MOR stimulation, which relaxes the smooth muscle in the gastrointestinal tract [32,33], making it one of the most common side effects of opioids. Our study suggests that morphine and hydromorphone are more likely to induce constipation. Previous research has extensively documented morphine-induced constipation [34,35,36], and the results of PCA in the current study reinforced morphine’s prominent association with this side effect. Conversely, switching to tapentadol may help reduce constipation incidence, making it a viable option for patients experiencing severe constipation.

#### 4.1.3. Somnolence

Somnolence [37], a central nervous system effect induced by opioids, was categorized in the high-incidence group, being particularly prominent with oxycodone and morphine. Previous studies [38,39,40] have similarly reported that oxycodone induces central side effects, such as somnolence and vomiting, relatively more frequently, consistent with our PCA outcomes. In contrast, hydromorphone appears to cause somnolence relatively less frequently, indicating a suitable option for cases where minimizing sedation is a priority [41,42].

#### 4.1.4. Dizziness

Dizziness [43] was also categorized in the high-incidence group, reflecting another central nervous system effect of opioids. In our study, PCA positioned dizziness alongside nausea and somnolence in the positive direction of the first principal component, suggesting a correlation with other central nervous system side effects. However, as only a few previous studies have explicitly focused on dizziness, further clinical research is needed to deepen our understanding of this side effect.

#### 4.1.5. Headache, Pruritus, Asthenia, Dry Mouth, and Hyperhidrosis

Headache, pruritus, asthenia, dry mouth, and hyperhidrosis were categorized in the low-incidence group during cluster analysis. However, hydromorphone and tapentadol have been noted to induce headaches more frequently [44], whereas morphine is associated with dry mouth [45] and hyperhidrosis [46]. Our findings support these findings and underscore the importance of close monitoring for these less common side effects in clinical practice.

### 4.2. Overall Implications from PCA

In the current study, PCA focused on the five high-frequency side effects, namely nausea, vomiting, constipation, somnolence, and dizziness, identified through cluster analysis. The first and second principal components explained approximately 75.1% of the data variance, with the first component distinguishing between central factors (nausea, vomiting, somnolence, and dizziness) and peripheral factors (constipation) while the second component further distinguishing vomiting and somnolence. This analysis allowed us to visualize the distinct risk profiles of each opioid. Morphine and hydromorphone were more strongly associated with constipation, fentanyl and tapentadol with nausea and vomiting, and oxycodone with somnolence (Figure 2).

### 4.3. Application in Opioid Switching

These findings provide valuable guidance for clinicians when considering opioid switching strategies. For instance, patients experiencing severe constipation may benefit from switching to tapentadol, whereas those suffering from pronounced nausea or vomiting may find relief by transitioning to opioids with a lower incidence of these side effects. The comprehensive side effect profiles revealed through our analysis can support the advancement of personalized medicine by optimizing opioid selection based on each patient’s specific clinical needs.

When transitioning from one opioid to another, a process often termed as “opioid rotation” or “opioid switching”, it is essential to maintain adequate analgesia while minimizing adverse effects. This typically involves using equianalgesic conversion to determine a starting dose for the new opioid, followed by careful titration. For instance, if a patient develops severe constipation while on morphine, then switching to tapentadol might reduce gastrointestinal side effects. However, the initial tapentadol dose should be selected to provide comparable pain relief, with subsequent dose adjustments based on the patient’s pain and adverse effect profiles.

As an example, we considered the case of a patient with advanced cancer-related pain who experienced intolerable constipation and suboptimal sedation control on morphine. After reviewing the incidence of side effects for other opioids, such as the lower risk of constipation associated with tapentadol, the clinician might opt to switch, ensuring that an appropriate equianalgesic dose is calculated. If the patient’s pain is managed appropriately and constipation improves following the switch, then the safety and effectiveness of the chosen strategy can be assumed. This example highlights how a comprehensive understanding of opioid side effect profiles, including the findings of SIDER-based analysis, can guide more personalized and effective pain management decisions.

### 4.4. Limitations

Several limitations of the present study should be noted. First, the SIDER database was last updated in 2015 [16], and the lack of newer findings potentially limits the generalizability of our results to current clinical practice. Furthermore, Kuhn et al. [16] reported that SIDER lacks incidence data for 39% of all drug-side effect pairs, hindering a complete understanding of the full spectrum of side effects. The SIDER website [15] also states that many medical concepts not directly representing side effects have been excluded, emphasizing the need for further data accumulation, including data from real-world pharmacovigilance databases such as FAERS and JADER. In addition, the aggregated data in SIDER may have been affected by reporting biases and inconsistent collection standards across different studies and package inserts. Thus, heterogeneity in study methodologies, such as variations in patient populations, dosing regimens, and routes of administration, may have influenced the reported incidence data.

Second, missing data affected our analysis. For instance, methadone was excluded because of insufficient incidence data, preventing a comprehensive comparison with the five included opioids. Similarly, certain side effects lacked robust data for one or more of these opioids, limiting the breadth of our assessment. Consequently, 10 side effects were selected based on the criterion that each adverse event has reported incidence data across all five target opioids. Although this criterion allowed us to conduct standardized comparisons, it led to the exclusion of other potentially relevant side effects. Moreover, although our method of standardizing and transforming the reported incidence data facilitated cross-drug comparisons, it did not fully account for confounding factors such as polypharmacy, individual genetic variability, and dose adjustments over time.

Despite these limitations, our analysis highlighted meaningful differences in the side effect profiles of commonly used strong opioids. By elucidating these distinctions, clinicians can make more informed decisions when considering opioid switching for patients experiencing intolerable side effects, ultimately improving pain management and patient outcomes.

## 5. Conclusions

In this study, we used the pharmaceutical data in the SIDER database to comprehensively analyze the incidence of side effects for five strong opioids commonly used in Japan: morphine, fentanyl, oxycodone, hydromorphone, and tapentadol. Through cluster analysis and PCA, we identified the distinct side effect profiles associated with each opioid. Although all the opioids commonly induced central side effects, such as nausea and vomiting, specific opioids differed in their propensity for causing constipation or somnolence, with some also exhibiting higher or lower incidences of headache and dry mouth. PCA further highlighted opioids particularly prone to causing constipation (morphine and hydromorphone) or nausea and vomiting (fentanyl and tapentadol), as well as those associated with somnolence (oxycodone). These insights can serve as objective indicators for opioid switching decisions, helping reduce adverse effects, such as constipation or nausea/vomiting, and enabling continued pain management in clinical settings.

## Data Availability

Data are contained within the article.

## Supplementary Materials

The following supporting information can be downloaded at: https://www.mdpi.com/article/10.3390/jcm14051410/s1, Table S1: Detailed Side Effects and Incidence Rates of Strong Opioids Based on Data from the SIDER Database.

## Abbreviations

The following abbreviations are used in this manuscript:

LLT	Lowest level term
PCA	Principal component analysis
PPPD	Persistent postural-perceptual dizziness
SIDER	Side effect resource
